# In the absence of cancer registry data, is it sensible to assess incidence using hospital separation records?

**DOI:** 10.1186/1475-9276-5-12

**Published:** 2006-10-06

**Authors:** Moyra E Brackley, Margaret J Penning, Mary L Lesperance

**Affiliations:** 1 Centre on Aging and Department of Anthropology, University of Victoria, PO Box 1700 STN CSC, Victoria BC V8W 2Y2 Canada; 2 Centre on Aging and Department of Sociology, University of Victoria, PO Box 1700 STN CSC, Victoria BC V8W 2Y2 Canada; 3 Department of Mathematics and Statistics, University of Victoria, PO Box 3045 STN CSC, Victoria BC V8W 3P4 Canada

## Abstract

**Background:**

Within the health literature, a major goal is to understand distribution of service utilisation by social location. Given equivalent access, differential incidence leads to an expectation of differential service utilisation. Cancer incidence is differentially distributed with respect to socioeconomic status. However, not all jurisdictions have incidence registries, and not all registries allow linkage with utilisation records. The British Columbia Linked Health Data resource allows such linkage. Consequently, we examine whether, in the absence of registry data, first hospitalisation can act as a proxy measure for incidence, and therefore as a measure of need for service.

**Methods:**

Data are drawn from the British Columbia Linked Health Data resource, and represent 100% of Vancouver Island Health Authority cancer registry and hospital records, 1990–1999. Hospital separations (discharges) with principal diagnosis ICD-9 codes 140–208 are included, as are registry records with ICDO-2 codes C00-C97. Non-melanoma skin cancer (173/C44) is excluded. Lung, colorectal, female breast, and prostate cancers are examined separately. We compare registry and hospital annual counts and age-sex distributions, and whether the same individuals are represented in both datasets. Sensitivity, specificity and predictive values are calculated, as is the kappa statistic for agreement. The registry is designated the gold standard.

**Results:**

For all cancers combined, first hospitalisation counts consistently overestimate registry incidence counts. From 1995–1999, there is no significant difference between registry and hospital counts for lung and colorectal cancer (p = 0.42 and p = 0.56, respectively). Age-sex distribution does not differ for colorectal cancer. Ten-year period sensitivity ranges from 73.0% for prostate cancer to 84.2% for colorectal cancer; ten-year positive predictive values range from 89.5% for female breast cancer to 79.35% for prostate cancer. Kappa values are consistently high.

**Conclusion:**

Claims and registry databases overlap with an appreciable proportion of the same individuals. First hospital separation may be considered a proxy for incidence with reference to colorectal cancer since 1995. However, to examine equity across cancer health services utilisation, it is optimal to have access to both hospital and registry files.

## Background

### Health service utilisation, equity and incidence

A major focus within the health service utilisation literature is to understand distribution of health and health services in the context of equity, that is, distribution with respect to social location indicators such as socioeconomic status, education and the like (e.g. [1-5]). It is relatively simple to describe health service utilisation over time; it is less simple to evaluate utilisation equity over time; the latter necessitates first understanding the distribution of need or requirement for service. That is, if utilisation rates of some service decrease over time within lower income quintiles, is this due to structural access inequity or is the requirement for those health services decreasing?

While more difficult with respect to home care or mental health services (where needs assessments vary depending on government policy), need for cancer-related health care services would seem to depend most fundamentally on incidence itself, a relatively objective state. Cancer incidence has been demonstrated in more than one jurisdiction to be differentially distributed with respect to a primary social locator, socioeconomic status (e.g. [6-11]), leading to an expectation of differential service utilisation across socioeconomic status, even given equivalent access to service.

However, not all jurisdictions maintain cancer incidence registries, nor do all existing registries allow linkage with utilisation records. How might researchers evaluate need for service in the absence of a cancer registry? First hospitalisation may vary with social location, and can be utilised as a measure of access to health services [12]. We examine here whether first hospitalisation may be used as a proxy for incidence, or need for service.

### Administrative claims data and disease registries

The relationship between disease registries and administrative claims data, and use of the latter for disease surveillance, has been investigated in several jurisdictions. With respect to diseases other than cancer, Goldacre and Roberts [13] assessed incidence of acute pancreatitis in Oxford (UK) on the basis of hospital admissions; they reasoned that this calculation would yield a good approximation of true incidence, as almost all people who receive a diagnosis of acute pancreatitis will be admitted to hospital. Hux et al. [14] recently validated a procedure using linked administrative data from Ontario and the Canadian Institute for Health Information (CIHI) to determine prevalence and incidence of diabetes in Ontario. They conclude that in a single-payer health care system, administrative data can provide a population-based opportunity to monitor chronic diseases. In contrast, Taylor [15] evaluated use of ICD-9 codes in administrative records to calculate incidence of bile duct injury during laparoscopic cholecystectomy in Ontario (see also comment by Marshall [16]). Taylor concluded that the clinical significance of the injury was not captured by the code without reference to a chart review, and therefore that the incidence of significant injury could not be calculated from the administrative records alone, which yielded an inaccurately high rate.

#### Do administrative data effectively complement cancer registries?

The question is not new. Morgan and Scott [17] assessed the performance of the (then) relatively new British Columbia Cancer Registry with respect to two factors: first, whether the registry missed an appreciable amount of hospital cancer separations (discharges); and second, whether using separations data would increase cancer incidence reporting. Female breast, bronchus and lung, and prostate cancer and acute leukemias were examined; the percent registered of those sampled from the separations data were 86.3%, 94.4%, 81.9% and 78.9% respectively. The authors concluded that cases missed by the registry on hospital separation likely would be collected on death registration, and that including these cases would shift registry counts upwards by a few percentage points.

Middleton et al. [18] evaluated hospital-only records for inclusion in the Northern Ireland Cancer Registry and conclude that discharge records constitute a valuable resource that nonetheless requires corroboration prior to inclusion in the registry. Without the discharge data, 11.5% of final database records would have been missing; however, without validation procedures, the registry would have increased inaccurately by 7.5% (from 18,703 to 20,229). In the United States, Wang et al. [19] compared claims data and a cancer registry in their ability to identify incident breast cancer cases in New Jersey, 1989–1991, amongst women registered with Medicaid, Medicare or the drug assistance program for the elderly (PAAD). Neither claims data nor the registry identified all incident cases; however, used in combination more than 90% of incident cases could be identified. The two data sources used in combination are most critical with respect to ascertaining subpopulations such as women under 65, minorities and the poor.

Penberthy et al. [20] examined the use of hospital discharge files to track cancer cases, noting that under-reporting remains a problem in central cancer registries. The study matched records from the Virginia Cancer Registry to those from state hospital discharge files for 1995. Only first admissions for an individual were included. Breast, cervical, colorectal, lung and prostate cancer cases were examined. A subset of cases was selected to assess positive predictive value (PPV) of the hospital discharge files judged by the inpatient medical record. Overall PPV was 94%, with 88% PPV for hospital-only records. This is one of the few studies where both discharge and registry datasets are evaluated simultaneously in reference to an independent gold standard. The authors conclude neither registry nor hospital files completely describe all incident cancer cases, and careful use of hospital discharge files to supplement the cancer registry would enhance cancer surveillance.

#### To what degree do registry and claims datasets describe the same individuals?

In New South Wales, Australia, McGeechan et al. [21] linked hospital and cancer registry datasets for invasive breast cancer cases in 1992. Ninety-three percent of records in the cancer registry were successfully linked to hospital records. Pollock and Vickers [22] examined colorectal, lung and female breast cancer registrations in the Thames Cancer Registry (UK) 1991–1994, to determine which records could be linked to hospital records. The study evaluated the extent to which death certificate only registrations could be reduced in the cancer registry. Sixty-six percent of cancer registry files found matches in the hospital dataset; for colorectal, lung and female breast cancer the numbers were, respectively, 72%, 62% and 65%. The authors conclude that cancer registry ascertainment trumps the accuracy of hospital files, which nonetheless can contribute to reducing the proportion of death certificate only records.

Stang et al. [23] compared agreement amongst linked Medicare (in-patient) records, cancer registry and death certificates in Massachusetts from 1986–1990. Kappa values for agreement were high between hospitalisation and death certificate data (κ = 0.70), and moderate between hospitalisation and cancer registry, and death certificates and cancer registry. With respect to specific cancers, the authors conclude that site-specific agreements among the databases were higher for colorectal and respiratory tract cancers as compared to breast and prostate cancers. Cooper et al. [24] calculated the sensitivity of Medicare claims data to identify cancer cases using Surveillance, Epidemiology and End Results (SEER) Program records, linked to hospital and other records in SEER-enrolled cities in the United States. SEER records were designated the gold standard; breast, colorectal, endometrial, lung, pancreatic and prostate cancers were included in the study. The authors conclude that Medicare claims yield acceptably high sensitivity (up to 94% depending on cancer type and range of records utilised) in reference to registry data. In contrast, Koroukian et al. [25] linked Ohio Medicaid Claims Data from 1997 and 1998 with corresponding years in the Ohio Cancer Incidence Surveillance System. The aim was to assess the ability of claims data to correctly identify incident breast cancers among women aged 40+, using the OCISS as the gold standard. Overall sensitivity was 68.7%, with overall PPV 43%.

#### Can claims data be used to calculate cancer incidence?

Toniolo et al. [26] estimated cancer incidence in a region of northern Italy from hospital discharge records, linking hospital to cancer registry records. Results indicated generally higher incidence rates calculated from hospital as compared to registry records. The authors attribute this difference to a number of factors, including the presence of prevalent cases, errors in coding and multiple hospital admissions. Nonetheless, they note that for some sites, such as male lung and colorectal cancers, there is near-equivalence for both count and age-sex distribution between the two data sources. Quality of information seemed lowest in the extreme age groups; the authors suggest this is due to lack of hospitalisation amongst older patients, and small sample fluctuations in the youngest age groups. They conclude that hospital files yield acceptable incidence estimates for more frequent cancers (here, esophageal, stomach, colorectal, pancreatic and lung cancers) using truncated age groups 35–74. Similarly, Huff et al. [27] report that hospital discharge data from the Chronic Disease Surveillance System in Maine for lung, cervical and female cancer consistently overestimated annual counts and disease rates in relation to cancer registry records. Prevalent cases may have been partially responsible for the annual count excess, but the difference persisted once records were linked across years to calculate aggregate period counts and rates. The proportion of 1987–1988 hospital discharge records that matched cancer registry data were 66%, 40% and 77% for lung, cervical and breast cancer respectively. The authors note that variation in admitting practises over time and area might have resulted in observed discrepancies between registry and hospital data, and that cases reported to the registry may have been incomplete.

In the absence of hospital-based cancer registries in France, Couris et al [28] proposed a system to utilise claims data to generate valid incidence measures. Similarly, Leung et al. [29] developed an algorithm to correctly identify incident breast cancer cases from women enrolled in a California (US) health maintenance organisation from 1994–1996; the authors conclude that claims data may be used to identify incident breast cancer cases. Finally, Pearson et al. [30] discuss the challenge of using multiple data sources to identify all those individuals, and only those individuals, with cancer. They conclude that problems with sensitivity and positive predictive value limit the usefulness of claims data for identifying incident cancer cases, and while the American cancer registry system is not comprehensive, cancer registries represent the best bet for incident case identification.

## Summary

Administrative claims databases are being used to study disease occurrence and distribution; these data are best used in conjunction with available disease registries. However, not all jurisdictions have cancer registries; nor, where there are registries, do these necessarily allow linkage with utilisation data. In addition, researchers do not always have access to all data sources. Huff et al. [27] state that "...hospital discharge data will be better suited to tracking disease occurrence where multiple admissions for a single individual may be accurately identified, variability in provider practise with respect to hospitalisation is minimal, and access to care is consistent" (p.81). These conditions are reasonably well met with respect to the data analysed here, given the provenance of the data source (below), and the provisions of the Canadian universal single-payer health care system (see also Hux et al. [14] above). In British Columbia, the British Columbia Linked Health Data (BCLHD) resource allows hospital separation records to be individually linked to cancer incidence records from the registry. This resource permits an assessment of the congruence of hospital separation and cancer incidence data. We examine here whether first separation can act as a proxy measure for incidence, and therefore as a measure of need for service. That is, if we hypothesize that first hospital cancer separation may be used as a proxy for cancer incidence, then this is a testable hypothesis within the British Columbia context.

## Methods

### Data source

Data were drawn from the British Columbia Linked Health Data (BCLHD) resource, an administrative health data repository for health services records included within the provincially-funded health services plan, for example, hospital separations, physician services, continuing care and other services, and including the British Columbia Cancer Registry (Chamberlayne et al. [31]). These data are accessible to researchers through the British Columbia Ministry of Health; the resource is compiled and distributed by the University of British Columbia Centre for Health Services and Policy Research (CHSPR) Health Information Development Unit [32]. CHSPR generates unique study identification numbers for individuals at the time of the data draw; these numbers are consistent across linkable datasets. Use of the BCLHD for this project was approved by BC Ministry of Health, BCLHD Data Stewards and the University of Victoria Human Research Ethics Committee.

The British Columbia Cancer Incidence Registry was established in 1969 and is maintained at the British Columbia Cancer Agency (BCCA). The registry records population-based cancer incidence and mortality, with associated demographic and personal information, for all residents of British Columbia [33]. Incident cancers are classified in accordance with International Classification of Diseases for Oncology-Second Revision (ICD0-2) codes. Multiple primary cancers attributable to a single individual are maintained as separate records. A number of sources contribute to the registry, including the Cancer Agency itself and hospitals; incidence records are also produced from death registrations.

Hospital separation records are generated each time an individual is discharged from hospital, whether treated as an in- or out-patient. Hospital files may include multiple separations attributed to a single individual. Up to sixteen diagnoses are included on each separation record, using International Classification of Diseases, Revision 9 (ICD-9) codes; the principal diagnosis is that deemed most responsible for the hospital stay. In the following analyses, where first separations are specified, these designate the initial appearance of a study identification number in the defined dataset.

The study utilised cancer registry and hospital separations datasets representing a 100% draw for the Vancouver Island Health Authority (VIHA) within British Columbia. This Health Authority includes Vancouver Island, the Gulf Islands and a section of coastal British Columbia. VIHA mid-year population size over the time period ranged from 588,441 in 1990 to 690,879 in 1999 [34].

Calendar years 1990 through 1999 are present for cancer registry data; fiscal years 1989–1990 through 1999–2000 for hospital data. Hospital records dated 1989 and 2000 were excluded from the analysis; separations then span the same time period as the registry.

Hospital separations with principal diagnoses indicating malignant neoplasms were included in the analyses, excluding non-melanoma skin cancers (ICD-9 173); similarly, non-melanoma skin cancer (ICDO-2 C44) was excluded from cancer registry records. Radiotherapy and chemotherapy hospital separations were excluded from the analysis as ICD-9 codes for radiotherapy and chemotherapy do not indicate type of cancer. Hence, using these codes from the hospital files for an individual's first occurrence would preclude comparing type of cancer coded by the hospital and by the registry. The effect of eliminating chemotherapy and radiotherapy codes is to permit a subsequent record for an individual, with a cancer code, to stand for the individual. Data are ultimately excluded only for those individuals who have *only *treatment-coded records.

Specific cancers examined are lung, colorectal, female breast, and prostate cancers, defined in accord with National Cancer Institute of Canada ICD-9 [35] and ICDO-2/3 [36] specifications. See Table 1 for codes used in the analyses.

**Table 1:** Classification codes.

**Category**	**Hospital separations : ICD-9**	**Cancer registry : ICDO-2**
All cancers	140–208, excluding 173	C00-C97, excluding C44
Lung cancer	162	C33-C34
Colorectal cancer	153–154, 159.0	C18-C21, C26.0
Female breast cancer	174	C50
Prostate cancer	185	C61

Cancers are defined in accord with the National Cancer Institute of Canada specifications [35,36]. Non-melanoma skin cancer is excluded from hospital and registry data; chemotherapy and radiotherapy representing first separation are excluded from the hospital records.

## Analysis

This paper assesses the extent to which administrative hospital separation records may be used to measure cancer incidence. At base, this is an exercise in counting individuals across age and sex through the study period. As hospitals are the main source of information for the cancer registry, we expect considerable overlap between the hospital separations and registry files. Since both registry and separations files for the region are 100% draws, it is possible as well to assess regions of exclusivity between the files. Lacking an independent gold standard, we designate registry data as the gold standard; the BC Cancer Registry reports 91.9% case ascertainment completeness for 2001 [33]. Table 2 outlines how individual study identification numbers distribute between registry and separations files. We examine whether annual first counts and age-sex distributions for hospital separations are significantly different from corresponding registry values.

**Table 2:** Distribution of unique study identification numbers between cancer registry and hospital separations.

**Study identification numbers**	**Cancer registry present**	**Cancer registry absent**	**Totals**
**Hospital separations present**	a	b	(a + b)
**Hospital separations absent**	c	d	(c + d)
**Total**	(a + c)	(b + d)	(a + b + c + d)

Notes.

1. Total unique study identification numbers = (a + b + c)

2. All study identification numbers from BCCA cancer incidence registry = (a + c)

3. All study identification numbers from hospital cancer separations = (a + b)

4. Shared study identification numbers between BCCA registry and hospital separations = a

5. Study identification numbers only in the BCCA registry = b

6. Study identification numbers only in hospital separations = c

7. Reference population used for annual calculations is the mid-year VIHA population = (a + b + c + d)

8. Count "d" represents the balance of the VIHA mid-year population appearing in neither registry nor separations records

9. In the case of the 1990–1999 aggregate file, the ten-year mean mid-year population is designated as the reference population; see discussion in text for effect of this assumption

10. The cancer registry is taken as the gold standard

Measures.

1. Similarity = [(a + b)/(a + c)]

2. Sensitivity = [a/(a + c)]

3. Specificity = [d/(b + d)]

4. Positive predictive value = [a/(a + b)]

5. Negative predictive value = [d/(c + d)]

More specifically, and annually, we count the following with respect to all cancers and specific cancers: 1) all registry cancers; 2) all hospital cancer separations; 3) unique study identification numbers in the registry files, and their corresponding frequencies; and 4) unique study identification numbers in the separations files, and their corresponding frequencies. We compare the annual count of unique study identification numbers in registry and hospital separations files using Pearson's chi-square test for goodness-of-fit.

**Figure 1:**
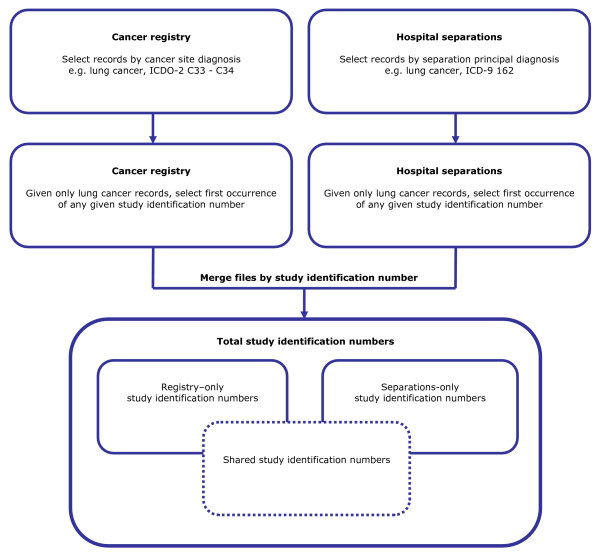
Procedure for merging registry and hospital files.

We then merge annual registry and separations files by study identification numbers, selecting for: 1) all cancer codes; 2) lung cancer only; 3) colorectal cancer only; 4) female breast cancer only; and 5) prostate cancer only. See Figure 1 for an outline of the merge procedure. We compare the degree to which the hospital separations report the same individuals, not simply the same count, as the registry files, by examining total unique study identification numbers, and their distribution between and within registry, separations and shared domains.

Counts and measures are calculated for annual files, and for a single file representing the entire ten-year study period (1990–1999). Total unique counts in the ten-year file differ from the sum of the annual counts. This is because individuals may be present either in registry or separations files more than once within a given year or across years, and for one or more cancer types. For example, in the registry, 2 – 3% of individuals included in the annual files, and about 6% of those in the ten-year aggregate file, record multiple incident cancers. For the annual count, an individual is included the first time they appear each year with respect to the target condition (e.g. lung cancer); similarly for the ten-year count, each individual is counted the first time they appear in the ten-year file, again with respect to the target condition.

We calculate the sensitivity of the separations files judged by the gold standard of the registry. We also assess specificity, and positive and negative predictive values (PPV, NPV), of the separations files (see Table 2 for definitions). We then calculate the kappa (κ) statistic [37,38] for measuring agreement beyond that expected by chance between the hospital separations and registry files.

For annual files, the mid-year population is the reference population. A difficulty with the ten-year period calculations is choosing the correct reference population. It is neither the mean annual population for the ten-year period nor the ten-year summed total population. The correct reference population would be all individuals present at any time over the ten-year period, a number between the mean and the summed total population, and is unknown. We use the ten-year mean population, choosing to err on the side of underestimation. The effect of this choice is as follows. Neither sensitivity nor positive predictive value are affected, as the crucial numbers for those calculations – the registry count, the hospital count and the distribution of cases between the two–are known independent of the reference population. Choosing the smaller reference population does affect specificity, negative predictive value and kappa; all three will be underestimated using this assumption.

Where registry and hospitalisation are both present but do not occur within the same calendar year, the ten-year merge will find the corresponding records, whereas annual files will miss the match. Consequently, we also calculate the kappa for agreement between diagnosis year and separation year amongst shared study identification numbers in the ten-year file.

Finally, if first hospital cancer separation count is acceptable as proxy incidence, then age-sex structure should be similar between registry and hospital files. Here we encounter a peculiar situation. We know the age-sex distribution of both hospital separations records and those registry records that successfully merged with the hospital separations files. We do not know the age-sex distribution of the cancer registry file itself, since neither sex nor age at diagnosis were available from the BCLHD cancer registry dataset [32]. Instead, and as an approximation, we use projected age-sex cancer incidence counts for the Vancouver Island Health Authority in 2003 [33], and compare 1999 first separations data to that projection. We also assess the age-sex distribution of successfully merged registry records compared to that of summed annual age-sex counts for first hospital separations. In both cases we employ Pearson's goodness-of-fit test, using expected proportions generated in the former case by the projected incidence counts, and in the latter case by first separations.

All data were maintained and analysed using SAS v8.0 [39]. Sensitivity, specificity, positive and negative predictive values and their confidence intervals were calculated using GraphPad Instat [40]; goodness-of-fit tests for distributions were calculated using GraphPad [41]. Probability values for goodness-of-fit tests for counts were calculated using S-Plus v6.2 [42]. Confidence intervals for registry counts (95% CI) were calculated with Pezzullo [43].

## Results and discussion

Study period total counts for the cancer registry and hospital separations are reported in Table 3. Approximately 82% of records in the VIHA registry dataset were contributed by hospital sources, with 6% of cases reported by the BC Cancer Agency itself, 3% by death registrations and 1% by other sources (including out of province cancer registries and private physicians); 8% were missing sources. Pathology was the primary method of confirmation, for 84% of records in the registry. All records in the VIHA cancer registry were associated with a VIHA postal code of residence. With respect to hospital separations, 90.8% of records were reported from hospitals within the Vancouver Island Health Authority, and 99.2% of unique study identification numbers in the hospital cancer separations dataset were associated with a VIHA postal code of residence at the time of separation.

**Table 3:** VIHA cancer registry and hospital cancer separations, count of all records.

**Registry**	**All Cancers**	**Lung**	**Colorectal**	**Female Breast**	**Prostate**
1990–1999	34,023	4,505	4,379	5,101	5,613
1990	2,830	388	392	415	496
1991	3,133	423	390	498	545
1992	3,241	457	415	459	574
1993	3,555	454	429	467	718
1994	3,271	408	461	495	532
1995	3,290	461	409	483	419
1996	3,510	469	446	568	529
1997	3,691	486	486	590	572
1998	3,627	460	475	557	559
1999	3,875	499	476	569	669

**Hospital**	**All Cancers**	**Lung**	**Colorectal**	**Female Breast**	**Prostate**

1990–1999	60,255	7,362	6,216	6,341	7,660
1990	6,260	857	610	578	942
1991	6,382	911	580	664	913
1992	6,432	895	573	660	1,024
1993	6,533	844	617	592	998
1994	5,892	643	625	631	719
1995	5,797	699	578	627	593
1996	5,733	685	580	682	584
1997	5,975	677	682	646	628
1998	5,636	576	665	619	620
1999	5,615	575	706	642	639

Cancer registry counts include all incident primary cancers reported to the registry, excluding C44, non-melanoma skin cancer. Hospital cancer separations include all separations with principal diagnoses as ICD-9 code 140 through 208, excluding 173, non-melanoma skin cancer, and excluding chemotherapy and radiotherapy separations.

Table 4 reports unique study identification numbers from registry and hospital datasets. The 34,023 registry records are generated by 31,976 individuals over the ten-year study period. The mean number of primary cancers per individual is 1.06 (standard deviation (SD) 0.26), the mode is one, and the range is between 1 and 4. The maximum number of incident cancers within any year reported for any one individual is three. The 60,255 cancer separations are generated by 31,203 individuals over the ten-year study period, with a mean of 1.93 (SD 1.55), mode of one, and a range from 1 to 34 separations. The maximum number of separations within any given year reported for any one individual is twenty-two.

**Table 4:** VIHA cancer registry and hospital cancer separations summary statistics.

**Registry**	**n**^1^	**mean**^2^	**SD**^3^	**minimum**^4^	**maximum**^5^
1990–1999	31,976	1.06	0.26	1	4
1990	2,780	1.02	0.13	1	2
1991	3,054	1.03	0.16	1	3
1992	3,179	1.02	0.14	1	3
1993	3,493	1.02	0.14	1	3
1994	3,210	1.02	0.14	1	2
1995	3,231	1.02	0.14	1	3
1996	3,423	1.03	0.17	1	3
1997	3,616	1.02	0.14	1	2
1998	3,538	1.03	0.16	1	3
1999	3,781	1.02	0.16	1	3

**Hospital**	**n**^1^	**mean**^2^	**SD**^3^	**minimum**^4^	**maximum**^5^

1990–1999	31,203	1.93	1.55	1	34
1990	4,031	1.55	1.10	1	22
1991	4,150	1.54	1.00	1	15
1992	4,252	1.51	0.93	1	12
1993	4,332	1.51	0.90	1	11
1994	4,010	1.47	0.87	1	13
1995	3,939	1.47	0.88	1	16
1996	3,947	1.45	0.91	1	15
1997	4,206	1.42	0.77	1	11
1998	4,067	1.39	0.77	1	15
1999	4,102	1.37	0.72	1	15

For both datasets, the sum of unique study identification numbers each year over the study period will exceed the all-years total, because repeat individuals may span a number of years. For example, in the registry there are 110 study identification numbers in the ten-year period with three primary cancers, but only 15 record all three within a single year.

^1 ^n total count of unique study identification numbers

^2 ^mean average number of records *per *unique study identification number

^3 ^SD standard deviation

^4 ^minimum minimum observed records *per *unique study identification number

^5 ^maximum maximum observed records *per *unique study identification number

**Figure 2:**
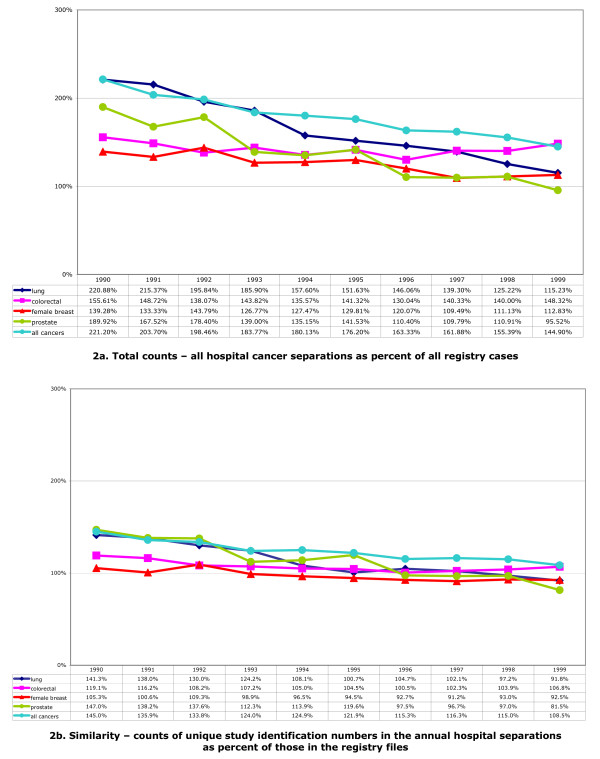
**Hospital cancer separations counts as percent of cancer registry counts, 1990–1999**. See Table 1 for cancer classifications codes included in the counts.

Figure 2 illustrates annual hospital counts as percent of annual registry counts for all cancers, and lung, colorectal, female breast and prostate cancers. Figure 2a depicts total counts, including multiple records attributed to single individuals. The curves are generated by the relationship between the number of incident cancer diagnoses and the pattern of initial and subsequent hospitalisations. Assuming a relationship between the registry and hospital records, where multiple hospitalisations characterise a cancer, the curve will lie above the 100% line; where the cancer does not always result in hospitalisation, the curve will dip below 100%. Actual values range from 221% (all cancers and lung cancer in 1990) to 95% (prostate cancer in 1999). Figure 2b represents annual first counts, and depicts similarity in counts for unique study identification numbers in the respective datasets. All values start the decade above 100%; similarity coalesces around 100% in the latter half of the decade.

**Figure 3:**
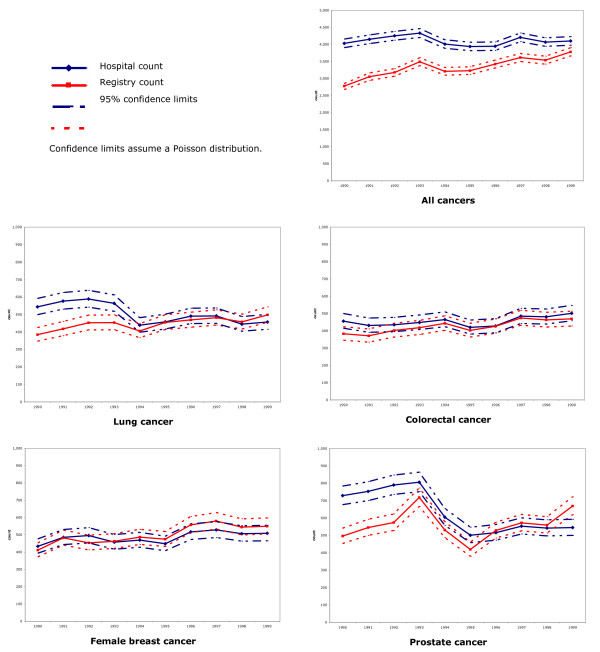
VIHA cancer registry and hospital cancer separations annual first counts.

Figure 3 illustrates annual counts for unique study identification numbers for the registry and hospital separations, with 95% confidence intervals for the registry counts. The hospital all-cancers count consistently overestimates the registry count over the study period. The same cannot be said of the individual cancers. Pearson chi-square goodness-of-fit tests across the entire ten-year study period indicate significant differences in annual counts between the datasets for all cancers and the specific cancers examined. However, using the sequence of counts for only the second half of the decade (1995–1999), these tests indicate annual counts for lung and colorectal cancer are not significantly different between registry and hospital files (p = 0.42 and p = 0.56, respectively); counts in the second half of the decade are significantly different for all cancers, and female breast and prostate cancer.

**Figure 4:**
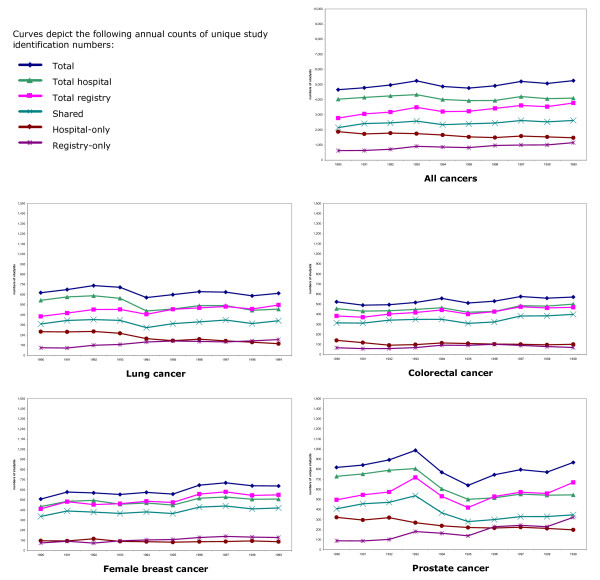
**VIHA cancer registry and hospital cancer separations: total, shared and restricted study identification numbers, 1990–1999**. Merge files annually by-cancer study identification number, 1990 through 1999

**Figure 5:**
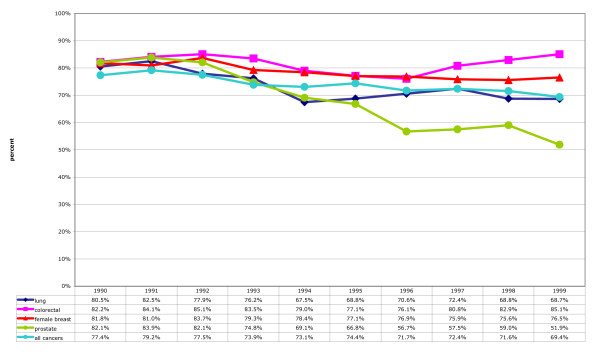
Sensitivity: percent of VIHA cancer registry annual count of unique study identification numbers represented by study identification numbers shared between registry and hospital cancer separations.

Figure 4 illustrates the results of merging registry and first cancer separations files by unique study identification number for all cancers and, separately, lung, colorectal, female breast and prostate cancer. There are six curves for each cancer: the total number of unique study identification numbers present across both registry and separations datasets; study identification numbers from the registry dataset, and the cancer separations dataset; study identification numbers present in the shared dataset, that is, successfully matched study identification numbers; and study identification numbers in the registry only, and in the separations file only. Figure 5 illustrates sensitivity between registry and first separations files, that is, the percent of registry unique study identification numbers shared annually with hospital separations files. Annual values for sensitivity for all cancers combined and specific cancers begin the decade clustered closely around 80%, and decline (except for colorectal cancer) and disperse thereafter. Sensitivity is greatest for colorectal and female breast cancer.

From the foregoing it will be clear that not all individuals in the cancer separations file are present in the registry, and *vice versa*; in addition it is clear from the sensitivity values that not all individuals are first hospitalised for a specific cancer during the same year in which their diagnosis is recorded in the registry.

Table 5 details ten-year period values for sensitivity and specificity, and positive and negative predictive values of separations to the registry gold standard; kappa values for agreement between registry and separations records; and, given the subset of study identification numbers shared between datasets over the aggregate ten year period, kappa values for agreement between year of diagnosis in the registry and year of separation from the hospital file. Except for sensitivity above, annual values for each of these measures are not shown; they are available on request to the authors.

**Table 5:** Sensitivity, specificity, positive predictive value, negative predictive value and agreement (kappa) for ten-year aggregate period 1990–1999.

	**All Cancers**	**Lung**	**Colorectal**	**Female Breast**	**Prostate**
**Sensitivity**	**78.58%**	**77.56%**	**84.22%**	**81.60%**	**73.00%**
lower 95% confidence limit (CL)	78.12%	76.27%	83.13%	80.48%	71.85%
upper 95% CL	79.04%	78.78%	85.34%	82.66%	74.12%

**Specificity**	**99.02%**	**99.88%**	**99.92%**	**99.86%**	**99.66%**
lower 95% CL	98.99%	99.87%	99.91%	99.84%	99.64%
upper 95% CL	99.05%	99.89%	99.93%	99.87%	99.68%

**Positive predictive value**	**80.53%**	**81.82%**	**87.07%**	**89.54%**	**79.35%**
lower 95% CL	80.10%	80.60%	85.96%	88.61%	78.26%
upper 95% CL	80.95%	82.97%	88.09%	90.44%	80.51%

**Negative predictive value**	**98.90%**	**99.85%**	**99.90%**	**99.72%**	**99.52%**
lower 95% CL	98.87%	99.84%	99.89%	99.71%	99.50%
upper 95% CL	98.93%	99.86%	99.91%	99.74%	99.54%

**Kappa (total)**	**0.79**	**0.80**	**0.86**	**0.85**	**0.76**
lower 95% CL	0.78	0.79	0.85	0.84	0.75
upper 95% CL	0.79	0.80	0.86	0.86	0.77

**Kappa (syear by dyear)**	**0.91**	**0.93**	**0.96**	**0.95**	**0.91**
lower 95% CL	0.91	0.92	0.95	0.95	0.91
upper 95% CL	0.92	0.94	0.97	0.96	0.92

Notes.

1. Cancer registry and hospital cancer separations ten-year aggregate files merged by study identification number, selecting for cancer type and then for first occurrence of study identification number.

2. For sensitivity, specificity, positive predictive value and negative predictive value, the cancer registry is designated the gold standard.

3. Kappa (total) measures agreement on study identification numbers between registry and separation records.

4. Kappa (syear by dyear) assesses agreement between separation year and diagnosis year for the subset of shared study identification numbers across the ten-year period.

5. Reference population used is the British Columbia midyear population size for both sexes, or single sex with respect to female breast and prostate cancer. See text for discussion of choice of reference population for ten-year period.

6. Source for VIHA population figures from 1990 through 1999: BC Stats 2004: .

7. Not shown: measures calculated by individual year. These are available on request.

Registry and separation records with a single study identification number that do not occur in the same year will be matched in the ten-year file. Ten-year sensitivity ranges from 73.0% for prostate cancer to 84.2% for colorectal cancer. Recall that hospitals are the main source for cancer diagnoses in the registry, for approximately 82% of records. All sensitivity values other than prostate cancer approach this level, while colorectal cancer exceeds it. If an individual is present in the hospital dataset, and if 82% of registry records originate from hospitals, why might sensitivity drop below this level?

In fact, using *all *first hospital separations for the study period, that is, including all principal diagnoses, not just cancer diagnoses, the sensitivity of the ten-year hospital file with respect to the cancer registry is 96.5% (95% CI 96.3%–96.7%). So, the first part of the explanation for lower than expected sensitivity lies with the fact that while nearly all individuals present in the cancer registry are also present in the complete hospital separations file, not all of them are hospitalised recording a principal diagnosis of cancer.

An additional part of the explanation will reside with the study period itself. Where separations-only study identification numbers represent registry cases recorded prior to 1990, that is, where they constitute prevalent cancers, they will not appear in the registry during the study period, and consequently their study identification numbers will fail to find a match. For example, approximately twenty-eight percent of both ten-year all cancers combined and prostate cancer, and 26% of colorectal cancer, study identification numbers found only in separations files occur in 1990. Hence, prevalent cases will account for some of the lower than expected sensitivity.

However, while separations-only prevalent cases do not appear in the numerator when calculating sensitivity, they also do not appear in the registry during the study period, so they do not contribute to the denominator. The final part of the explanation resides with the group of study identification numbers that appear in the registry (so they are counted in the denominator) but do not appear in the separations files. These represent individuals for whom no hospital cancer separations occur, but who were registered with a primary cancer during the study period. 54% of ten-year aggregate registry-only records are 'other' cancers; lung, colorectal, female breast and prostate cancer contribute 10%, 7%, 11% and 18%, respectively, to the total number of registry-only study identification numbers. The distribution for the full ten-year registry dataset for 'other' and specific cancers is, respectively, 43%, 13%, 12%, 15% and 17%. There is a significant difference between the registry-only distribution of cancer types and the expected distribution from the full registry dataset (chi-square, p < 0.0001); 'other' cancers are over-represented amongst registry-only records. It might be informative to examine the age-sex distribution of the registry-only group, for example, to see whether they distribute differently from the shared registry-separations group, but as above, neither age nor sex is available for registry-only study identification numbers.

Ten-year specificity and negative predictive vales are uniformly high. This is in large part attributable to the size of the VIHA mid-year reference population and the relative rarity of a cancer diagnosis. Positive predictive value is variable, and consistently higher in the ten-year file than annual files across all and specific cancers; it ranges from 89.5% for female breast cancer to 79.35% for prostate cancer.

Kappa values for agreement on the presence of study identification number between registry and separations files range from 0.76, for prostate cancer, to 0.86, for colorectal cancer. The highest agreement is achieved with colorectal and female breast cancer, which fall in the 'almost perfect' range [37]; the remaining cancers, including all cancers combined, are represented by kappa values indicating 'substantial' agreement.

Kappa values for agreement between diagnosis year and separation year among those study identification numbers shared between registry and separations files are all above 0.90, in the upper half of the range described by Landis and Koch [37] as almost perfect. Where the two years are not identical, hospital separations occur primarily in the year immediately following registry diagnosis year. For example, for the all-cancers file in diagnosis year 1995, 92% of 2,429 cases were hospitalised in the same year, 3% were hospitalised prior to 1995, 4% in 1996, and the remaining 2% were hospitalised from 1997–1999. For colorectal cancer, of 317 shared study identification numbers reported in the registry in 1995, 96% are reported in the separations file in that year, while 3% are reported with separations in 1996. Of the two remaining shared cases, one separated from hospital in 1994 and one in 1997.

As above, we do not have access to registry age-sex structure from the BCLHD resource. Therefore, we compared first separations for 1999 to BCCA projections for VIHA incident cancer cases in 2003 [33]. Relative to the BCCA projection, first separation distributions by sex are not significantly different from the projection for all cancers combined, or colorectal cancer; lung cancer separations distribution by sex is significantly different from the projection with fewer males, and more females, than expected (p = 0.02). Only one sex is relevant for female breast and prostate cancer.

Age distributions are not significantly different for colorectal, female breast or prostate cancers for either sex, and for females for lung cancer. Both sexes for all cancers combined, and males for lung cancer, yield significantly different distributions between the hospital separations and incidence count projections (all cancers, males p = 0.0009, all cancers females p = 0.0001, lung cancer males p = 0.0045), with a similar pattern of more than expected separations among 60–79 year olds and fewer than expected separations among those 80+ years of age.

Finally, we examine ten-year summed annual counts for successfully merged registry records compared with those for first separations. There is no significant difference with respect to distribution by sex amongst lung and colorectal cancers (and, again, only one sex is relevant for female breast and prostate cancer). The all-cancers count indicates fewer males and more females than expected amongst successful registry merged records (p = 0.0001).

Among these merged data, there is no significant difference in age distribution for lung, colorectal or female breast cancer. All cancers combined yield significant results (males p = 0.0001, females p = 0.0012) with more merged registry records than expected from the hospital distribution amongst 60–79 year olds, and fewer records than expected amongst those 80+ years of age. Prostate cancer age distributions are significantly different (p < 0.0001), with a similar pattern.

## Conclusion

This paper contributes to literature addressing the utility of administrative claims data in health service utilisation research, with specific reference to equity, and the question of congruence between administrative claims data and disease registry records. We ask whether first hospital cancer separations may be used as a proxy measure for cancer incidence, using the registry file as the gold standard.

While these data are a sample of the provincial population, it is important to note that within the confines of the Vancouver Island Health Authority these data are not samples. Excluding administrative and recording error, they represent all events occurring in the study period. As such, differences between counts or distributions are true differences between populations during this period. We use statistics with associated confidence intervals and probability values to define the area within which a true difference is unremarkable, and outside which such a difference is noteworthy.

Where total counts and age-sex distributions are not significantly different, it follows that rates calculated from those figures will be acceptably equivalent. Does this hold in the present case? Goodness-of-fit tests across the study period (1990–1999) indicate significant differences in annual counts for all cancers and the specific cancers examined; however, comparison of counts from 1995 through 1999 yield non-significant differences between registry and separations counts for both lung and colorectal cancer. Distribution by sex between first separations count and projected registry counts indicate no significant differences between all-cancers and colorectal cancer (and the comparison is not applicable for single-sex cancers); age distributions do not differ for (female) lung, colorectal, female breast or prostate cancer. Colorectal cancer during the second half of the decade is the only case where there is no significant difference in count and age-sex distribution between hospital and registry datasets.

Do the two datasets describe the same individuals? Sensitivity values are highest for colorectal and female breast cancer, followed by all cancers combined, lung and then prostate cancer; positive predictive values follow a similar pattern. Reasons for lower than expected values of sensitivity include prevalent cases, that is, separations-only records, particularly in the early years of the study period, and the set of registry-only records not present in the separations files. This latter group will include both those individuals diagnosed but not hospitalised, and those who may have been hospitalised, but not with a cancer principal diagnosis coded on the separation record. "Other" cancers are over-represented amongst this group. We cannot comment on whether the registry-only dataset has a different age-sex distribution from those registry records shared with hospital separations, as this information is not available to us at present. However, it would appear that the successfully merged registry records, shared between registry and separations datasets, for lung, colorectal and female breast cancer do not represent different age-sex distributions from first hospital separations. Lastly, values for systematic agreement calculated by kappa indicate substantial to almost perfect agreement between registry and hospital files; agreement between year of diagnosis and separation for individuals shared between registry and separation files is consistently above 0.90, in the almost perfect range.

In summary, our findings suggest that in spite of the apparent similarity in count for each of the specific cancers examined, first separations are not an acceptable proxy for most cancer incidence counts, using the cancer registry as the gold standard. However, the hospital separations file acceptably approximates both registry count and age-sex distribution for colorectal cancer from 1995 through 1999. Additional research may generate correction factors to translate lung and female breast cancer first separations to valid incidence measures. If hospital separations were regarded as a screening tool for the registry, they would fail to be acceptable, given low sensitivity (range 73%–84%), and despite high specificity (>99%). Ten-year period positive predictive values are all above 80% (save prostate cancer, which falls just short of that figure); but do not extend above 90%.

This analysis has several limitations. First, we did not have access to an independent gold standard (for example, see [20]), and this means that the complete set of unique study identification numbers was not included in the sensitivity analysis. As a result, we do not know whether our results and conclusions would shift given a third-party gold standard. As well, the age-sex comparison was accomplished only obliquely, as these data were not available from BCLHD resource cancer registry datasets. In addition, for ten-year summary measures, the correct reference population is unknown; however the population used was chosen to underestimate specific measures, as discussed above. Finally, these results are calculated from data generated by a system with universal health care and first-world technology. Conclusions likely would not apply in jurisdictions where access to care is limited and/or treatment or admissions to hospital proceed in a systematically different manner.

Claims and registry databases overlap with an appreciable proportion of the same cases. However, there are also areas of exclusivity, that is, separations-only and registry-only cases. Even if only because of these exclusive cases, administrative data may complement but cannot replace the registry. Thus, for analysis of equity across cancer health services utilisation, it is optimal to have access to both hospital and registry files. Using the British Columbia Linked Health Data resource, first separation may be considered a proxy for incidence with reference to colorectal cancer since 1995. However, as discussed in the introduction, first separation itself may be examined as an indicator of access to cancer-related health services where incidence remains the primary indicator of need.

## Competing interests

The author(s) declare that they have no competing interests.

## Authors' contributions

MJP conceived and initiated our studies using the BCLHD resource to investigate health system change and its effects on equity; MEB specifically delineated cancer hospitalizations as an area of analysis. MJP. MEB and MLL all contributed to the analytical and statistical strategy. MEB managed the data, performed the statistical analysis and drafted the manuscript. All three authors edited and approved the final manuscript.
